# The chiropractic profession in Norway 2011

**DOI:** 10.1186/s12998-014-0044-5

**Published:** 2014-12-08

**Authors:** Ole C Kvammen, Charlotte Leboeuf-Yde

**Affiliations:** Private practice, Fokus Helse og Trening, Aagaards pl. 5, Sandefjord, 3211 Norway; Research Department, Spine Centre of Southern Denmark, Hospital Lillebaelt Middelfart, Institute of Regional Health Research, University of Southern Denmark, Odense, Denmark

## Abstract

**Background:**

The chiropractic profession in Norway has increased five-fold in the last two decades. As there is no academic graduate program in Norway, all chiropractors have been trained outside of Norway, in either Europe, America or Australia. This might have given Norwegian chiropractors heterogenic characteristics concerning practice routines and clinical settings. However, little is known about what characterizes this profession and how it compares to other chiropractic professions in Europe. The aim of this survey was to describe major characteristics of the chiropractic profession in Norway.

**Method:**

Two surveys were distributed to all 530 registered chiropractors in Norway in 2011. One survey was for all chiropractors (Survey 1) and the other for clinic owners (Survey 2). Results have been reported as tables and as approximate percentages in the text for ease of reading.

**Result:**

Response rates were 61% (Survey 1, N = 320) and 71% (Survey 2, N = 217). More than two-thirds of the chiropractors in Norway had been in practice for under a decade. Only one in four chiropractors worked in solo practice and the majority shared premises with at least one colleague, typically at least one physiotherapist and one additional health practitioner. Today, only one in five clinics possessed radiologic equipment and one in ten had access to diagnostic ultrasound equipment. The majority of the chiropractors reported to apply mainly similar treatment modalities. More than 90% reported to use manipulation techniques on most patients, with soft tissue techniques and exercise modalities being almost as common. More than 3/4 of the profession reported that their clinical practice was in accordance with available clinical guidelines and about one third were positive about participating in future clinical research.

**Conclusion:**

The Norwegian chiropractic profession is relatively young and members report being satisfied with their work conditions. There is a clear difference from the earlier practice pattern in that intra- and inter-professional collaboration is more common and it is considered desirable. The profession seems to follow the modern trends in evidence-based practice by using X-rays more sparingly than previously, adhering to guidelines and being positive about research.

## Introduction

Chiropractors all over the world practise under different legislations and circumstances. In Norway, the first chiropractor started his practice in 1922 [1] and the Norwegian Chiropractors’ Association was established in 1935, consisting of 20 individuals [2]. At the time, chiropractors practised under the so-called quackery law.

Legislation for chiropractors in Norway was introduced in 1989 [3]. Between 1974 and 2006, partial reimbursement was possible only if the patient had been referred by a medical practitioner. Since 2006, however, the profession has been authorized to practise as primary contact practitioners and their patients qualify for partial reimbursement from the national health care system [4]. With the new authorization, chiropractors are able to provide sick-leave and they can refer patients directly to radiological procedures or to other medical specialists for further assessment.

Today (2014), the chiropractic profession consists of approximately 650 individuals, of which the national association organizes more than 90%. The ratio of chiropractor/number of inhabitants is very high in Norway and according to records from the European Chiropractic Union. Norway and Lichtenstein are the European countries with the highest accessibility of chiropractors. In fact, the number of authorized chiropractors in Norway has increased five -fold in the last two decades. In addition, it is estimated that approximately 300 young Norwegians are enrolled in chiropractic graduate programs in various parts of the world. Hence, in 2020 the profession is estimated to consist of at least 1000 individuals.

This newly acquired favourable status as an officially recognized health profession is probably one of the reasons for the strong recruitment of chiropractic graduates in recent decades. However, this high number may seem puzzling, as there is no academic undergraduate institution for chiropractors in Norway. Although the high costs related to graduate programs abroad might diminish the interest for this type of education, this is probably offset by the fact that the Norwegian state offers beneficial educational loans and funding for foreign education and has a firm tradition of supporting and facilitating international education.

Easy access to care is obviously a good thing. However, the diversity of educational institutions that these chiropractors have attended could also result in an unacceptable heterogeneity of the profession. The fact that all chiropractors have a foreign educational background may result in a less successful integration of the profession into the Norwegian health care system. This might be due to both cultural and language differences between the country where the training was received and the country of practice, resulting in variations in practice routines and clinical assessment procedures.

However, little is known about the Norwegian chiropractic profession. Previous publications have reported mainly demographic data [5–12]. Modern concepts in relation to primary health care practice are those of co-operation, communication and multidisciplinary settings. It is not known if chiropractors follow these trends, or if many still practice in relative isolation in solo practice, as they did a few decades ago [13].

Other concerns could be that an overproduction of chiropractors may lead to some negative consequences, such as a tendency towards over-servicing, unsuitable “additional” procedures, reduced income and even unemployment. In order to learn more about current practice of Norwegian chiropractors two surveys were carried out: one aimed at all chiropractors in Norway and the other one for the clinic owners. The aim of this report is to describe major characteristics of the chiropractic profession in Norway, specifically to describe their clinical settings and practice routines, satisfaction with work and to study evidence of inter-professional collaboration, using data from these two surveys. In addition, the aim is to describe attitudes towards research participation and clinical guidelines.

## Methods

An invitation was sent to all 530 members of the Norwegian Chiropractors’ Association with a registered e-mail address in May 2011. The invitation contained a description of the two surveys and consent to participate to be signed. All chiropractors were asked to complete Survey 1 regarding practice routines. Clinic owners were asked also to answer Survey 2 regarding clinic settings and clinic routines.

Reminders were sent weekly until the end of data collection in August 2011. The invitation and reminders were administered by secretaries at the Norwegian Chiropractors’ Association.

These two surveys were largely based on two similar surveys of Danish chiropractors conducted in 2010, designed by researchers at the Nordic Institute of Clinical Chiropractic and Biomechanics (NIKKB Odense, Denmark). Permission to use this survey instrument was given by the administration of NIKKB.

Additional questions added to the Danish survey included some questions concerning compliance to the present national clinical guidelines for low back pain in Norway, and opinions on and attitudes to practice routines.

Data were collected in the open online survey database “Lime-survey”. As this was done anonymously, it was not possible to identify or compare responders and non-responders. The survey software did not permit more than one survey response. As the survey was anonymous and no track of participants was possible, repeated reminders to participate in the survey were sent regardless of previous participation.

As no personal data were collected, and all data collected anonymously, approval from the Regional Committee for Medical and Health Research Ethics was not needed.

Analysis was performed using the statistical software Analyze-It®. Obvious errors were amended manually when the correct information could be rationally extrapolated. Some responses were only partially completed. Such missing data have been labeled “no-responders” in the results section.

## Results

The response rates for Survey 1 (all chiropractors) and Survey 2 (clinic owners) were 61%(N = 320/530) and 71%(N = 217/306), respectively.

To determine the representativeness of the two study samples, comparisons were made between the responders and all members of the Norwegian Chiropractors’ Association in regard to sex and birthdate for Survey 1 and in regard to geographical distribution for Survey 2. In Survey 1 the representation of male responders was 64%, compared to 65% in the target population. With regard to age, there was a slight over-representation among the responders in Survey 1 of those below 40 years of age; but overall, all age groups were well represented. All counties were represented among the responders similar to the target population.

Results from both surveys have been reported below, grouped under separate topics and not by survey, in such a way that the profile of the chiropractic profession can best be described. In order to make the reading and comprehension easier, percentages have often been rounded off to the nearest “larger” figure in the text; i.e. 32.2% would be reported as “1/3” or as “about thirty percent” and 18.7% as “1/5” or “about 20 percent”. Exact estimates are available from the authors on request.

### Demographic data

Among the chiropractors who responded to the survey, 64% were male with the median and mean age of 36 and 38 years respectively and with 70% being below 40 years of age. Almost 4/5 had been in practice for less than 15 years, and only 8% had been in practice for more than 25 years (1% non-responders). The majority were trained in Europe; approximately 40% in the UK and 20% in Denmark; the others in North America (30%) or in Australia (10%) (2% non-responders).

None reported to have a PhD degree, although 2% reported to be involved in PhD programs. In addition, close to 50% reported to have some kind of additional higher education background: 7% at Master’s level (primarily a Master’s degree in Sports) and 18% at Bachelor level. The most frequently reported professional backgrounds were physiotherapy (5%) and nursing (1%) (53% non-responders).

### Clinical settings

Almost all chiropractors (97%) worked in private practice settings (3% non-responders). More than half of the clinics were located in an office building, adapted to wheelchair users, with access to an elevator, and in a building with other health practitioners, whereas only 20% practised in small, separate buildings (6% non-responders).

The majority (61%) had more than 4 treatment rooms in the clinic and therefore often practised together with other health practitioners (1% non-responders). In contrast, about one in four practised in solo practices. About 3/4 of the clinics consisted of more than one chiropractor; almost half of the clinics included at least one physiotherapist and one additional health practitioner (usually a massage therapist), but only 10% of the clinics reported to have a general practitioner linked to the clinic (0% non-responders).

About 1/3 of the clinics had access to large, separate exercise facilities or smaller exercise equipment (2/3), but imaging facilities were not common, as only 1/5 had their own X-ray equipment and 12% had diagnostic ultrasound. A mere 1% had magnetic resonance imaging facilities within their clinic setting (6% non-responders).

### Practice routines

Among all the chiropractors, one third reported the total weekly workload to be 30–37 hours and another third reported it to be 38 – 45 hours. A further 1/5 reported to work for more than 45 hours per week (3% non-responders). As 38 hours per week is considered full time office hours in Norway, this means that about half of chiropractors reported to work more than full time), whereas less than 10% worked for less than 25 hours per week. The majority of chiropractors (60%) reported to attend to 15 – 20 patients per day. Eighty percent reported spending 30–49 minutes on a first consultation and 60% spent 15–20 minutes on a regular consultation (2% non-responders).

The most common treatment modalities, i.e. those used on more than 50% of patients were reported to be: manipulation (97%), soft tissue techniques (80%) and instructions on home exercises (67%). More detailed information is provided in Table 1.

**Table 1:** **Treatment modalities reported to be used in a survey on Norwegian chiropractors (N = 320)**

**Manual techniques**	**Applied on the majority of patients (>50%)**	**Applied on minority of patients (≤50%)**	**No response (%)**
Activator	6.3	81.6	12.2
Traction	21.3	69.7	9.1
Mobilisation	21.6	70.6	7.8
Manipulation (extremities)	43.4	52.8	3.8
Manipulation (spine)	96.9	0.3	3.1
Soft tissue techniques			
Kinesio taping	3.8	81.9	14.4
Graston	5.6	77.2	17.2
Anatomy trains/soft tissue	12.2	69.1	18.8
Active release technique (ART)	20.9	66.9	12.2
Stretching	48.1	45.6	6.3
Triggerpoint	80.3	16.3	3.4
Exercise interventions			
Direct instruction exercise equipment	2.5	82.5	15.0
Direct instruction (Pilates/Bosu etc.)	20.0	66.3	13.8
Instruction home exercise	66.9	28.8	4.4
Advise/Cognitive modalities			
Cognitive techniques	10.9	77.2	11.9
Advise diet or supplements	25.9	68.4	5.6
Advise behaviour	42.5	49.7	7.8
Advise ergonomics	52.2	43.8	4.1
Additional modalities			
Sacro occipital technique (SOT)	3.1	82.8	14.1
Acupuncture/dry needling	17.8	68.8	13.4

The numbers in the table refer to the percentage % of chiropractors reporting if interventions/techniques were used more commonly (“Applied on the majority of their patients”), or less commonly (“Applied on the minority of the patients”).

In addition to instruction on home exercises, half of the chiropractors reported to refer patients to physiotherapists either within or outside the clinic. However, only one in five clinics offered some type of group exercise.

The use of X-rays was infrequent, either through referral to hospitals or private centres or (less commonly) within the clinics themselves. However, only 21% of the clinics reported to use radiological imaging on a daily basis. The questions in the survey allowed only one answer per category which might give rise to some under-reporting. For instance, it could be that chiropractors with X-ray imaging facilities in the clinic were still referring patients for more complex imaging at hospitals.

Full spine radiological imaging on a frequent basis (every day or week) was reported by almost 10%, either performed in their own clinic or by referral. The majority of chiropractors (62%) never referred for or reported taking full spine X-ray at all. Referral for MR imaging of patients was reported by more than three-quarters to occur at least every month. Referrals for ultrasound imaging were not as common as for X-ray and MR imaging (7–25% non-responders, several answers possible).

### Interdisciplinary collaboration

One in five reported to have interdisciplinary meetings at least every month. The most common health personnel to be engaged in such meetings were physiotherapists and massage therapists, in contrast to regular (monthly) meetings with GPs, which was reported to occur by only 20% of these (24% non-responders).

With regard to referrals, 3/4 of the chiropractors reported to refer patients to physiotherapists every month (as formal referral) and half of all chiropractors referred patients to orthopaedic specialists, 1/3 to neurologists, and 1/6 to rheumatologists, at least every month (4–12% non-responders, several answers possible). Communications to other health personnel were either written or verbal and for most occurred less than 5 times per week (6–22% non-responders, several answers possible). Chiropractors reported to engage infrequently with certain health personnel such as nutritional therapists and manual therapists. The vast majority (86%) reported the desire to have stronger and better cooperation with other health professions (6% non-responders).

### Patient demographics

About half of the chiropractors reported both that less than 10% of their patients were referred from general practitioners and other health personnel. Almost 4/5 reported that less than 10% of their patients were below 10 years of age. In addition, as many as 3/5 reported that between 10-50% of their patients had problems unrelated to the spine (5-7% non-responders, several answers possible).

### Attitudes to and knowledge of clinical guidelines

The majority (80%) of chiropractors reported that they practised in accordance with the Norwegian clinical guidelines for low back pain [14]. When participants were asked about specific guideline recommendations, most were able to recognize seven out of ten as being correctly cited (Table 2). In general, they also agreed with these recommendations.

**Table 2:** **Knowledge among Norwegian chiropractors about recent Norwegian clinical guidelines on low back pain reported in a survey on Norwegian chiropractors (N = 320)**

**Do you believe the following statements to correspond to recommendations in the Norwegian guidelines of low back pain?**					
**Statements**		**Yes (%)**	**No (%)**	**Don’t know (%)**	**No response (%)**
1.	Patients with non-specific low back pain and “green flags” have a good prognosis	65.6	4.7	4.4	25.3
2.	Patients with “red flags” could have possible pathology and should be further assessed	75.9	1.6	2.2	20.3
***3.***	***Radiological examination is recommended for patients with acute, subacute or chronic low back pain with no “red flags”***	19.7	50.9	4.7	24.7
4.	For acute, sub-acute and chronic low back pain patients; advise activity	73.8	1.9	2.8	21.6
**5.**	***Low back pain patients with nerve-root affection are advised NOT to be active if in pain***	28.8	37.2	5.6	28.4
6.	Surgery is recommended for patients with cauda equina and/or paralysis	74.7	1.3	2.2	21.9
7.	For non-specific low back pain patients manipulation is recommended, but scientific evidence is low	63.1	8.1	5.0	23.8
8.	Manipulation can be applied for patients with long-lasting low back pain with nerve-root affect.	39.4	29.1	6.3	25.3
9.	“Yellow flags” could be described as patients with e.g.an emotional/ psychological risk profile	68.1	3.1	4.1	24.7
10.	“Red flags” could be described as patients with e.g. morning stiffness, trauma history, or cancer	69.1	3.1	4.1	23.8

The statements in this table correspond to the recommendations given in the Norwegian guidelines (i.e. correct statements), in contrast to the two statements in bold text that do not correspond to the guidelines (i.e. incorrect statements).

### Clinical research participation

Only 5% were involved in Master’s programs and 4% were engaged in various research projects. However, almost 1/3 reported to be willing to participate in clinical research projects depending on the demands on their resources (e.g. extra time in relation to data collection) and compensation (economic or merit). Ten percent reported to be positive about participating in research-related projects regardless the circumstances (8 - 15% non-responders, several answers possible).

### Work satisfaction

Almost all chiropractors (90%) reported to be content with their choice of profession. Furthermore, the greater majority expressed that they had sufficient professional challenges and felt that their income reflected their workload. Nevertheless, more than half were uncertain or negative with regard to working conditions in the future (5-8% non-responders, several answers possible).

## Discussion

### Short summary

We performed two web-based surveys among registered chiropractors in Norway, members of the national chiropractic association. The results from these studies showed that the majority of Norwegian chiropractors are young and that they graduated relatively recently from a European school. Almost all work in private practice, with several health practitioners in the clinic and most of them work full time. Chiropractors’ treatment modalities vary to some extent, but in general, manipulation, soft tissue techniques and exercise advice are most commonly used. Although interdisciplinary cooperation is common with regard to communication, this applies to some health professions more than others, such as physiotherapists (most common) versus medical specialists (least common). Chiropractors appear to recognize, mainly agree with and practice according to a recent Norwegian guideline for back problems [14]. They have, in general, a positive attitude towards research and they are satisfied with their work, although there was some apprehension in relation to the future of the profession.

### Methodological considerations

The two surveys had a response rate of 61% and 71%, respectively. This raises some questions as to how representative the responses were in relation to all chiropractors in Norway. However, when demographic data from responders were compared with all the members of the Norwegian Chiropractors’ Association (NCA) (representing more than 90% of all chiropractors in Norway [15]), it was found to be very similar in relation to sex, age and geography.

One of the limitations of these two surveys was that no preparatory pilot study was performed. A pilot study may have revealed some weaknesses in the questionnaire allowing for the creation of a more user-friendly survey instrument, which in turn could have resulted in a lower rate of no response in some of the more complex questions. However, the major parts of the two surveys were based on a previous Danish survey [16], designed by experienced researchers, which could be considered as a test of questions and response alternatives.

In addition, one of the major limitations of the survey was the design itself. Data were primarily self-reported and not based on actual counts in patient files or appointment books, nor were they collected with clinical monitoring procedures. The result of this is that there was probably a higher response rate than there would have been with a more cumbersome data collection technique, but some information provided by the chiropractors would be subjective rather than clearly objective. This lack of precise information made it suitable to report the results in a general way, rather than reporting specific and detailed conclusions.

Some questions had quite a few non-responders. Nevertheless, the proportion of these was always taken into account in the analyses.

### Discussion of results

In the past, chiropractors worked almost exclusively as solo practitioners and in private practice. Today, more practitioners work in groups, together with other chiropractors and/or with other health practitioners [16,17]. This was clearly seen in our study. Still, only very few are employed in hospitals and research positions. The latter is hardly surprising, as there is presently no obvious career path for academic chiropractors, there being no chiropractic university-based (or other) undergraduate program within the country.

Despite the high density of chiropractors in Norway, as compared to other countries, only a few chiropractors reported working part time and none of them were registered as being unemployed. This aspect needs to be closely monitored in the near future as it is anticipated that the number of practitioners will increase considerably in the future.

### Practice routines

A wide variety of treatment modalities was reported among the chiropractors. Three major approaches were noted to be used by the majority of the chiropractors. These were: manipulation techniques, soft tissue techniques (mainly trigger point and stretching) and instruction/advice on exercise. This is not surprising as these findings probably best describe the activities of a chiropractor [16–23]. Techniques that are not recommended by the clinical guidelines [14,24–27] were not widely used.

Three decades ago almost three-quarters of all new patients presenting with LBP were assessed with radiological procedures in Norway [13], often as a precautionary measure. However, recent guidelines [14] have established clear criteria for when radiologic examinations are acceptable, with the result that there has been a considerable reduction in its use, which is also a similar trend in other countries [28]. As a consequence, today only a few clinics have their own radiological equipment. It could be argued that this is mainly for economic reasons, as modern X-ray equipment is an expensive investment and carries high maintenance costs. However, this study also revealed that there was only a modest degree of referrals for X-rays, so it is evident that chiropractors approve of this modern approach to X-ray examinations.

It is encouraging to see that some chiropractors have started using diagnostic ultrasound, which presents no harm (no radiation, no pain) to the patient. Further, it allows for dynamic examinations of muscles and tendons, allowing perhaps for an increased scope of practice.

### Interdisciplinary collaboration

Recent guidelines advise a multidisciplinary approach to the management of long-lasting musculoskeletal problems [14,25]. The findings in these surveys show that only the minority work in solo practices. This is in contrast to some other countries where about half of the profession were working in solo practices [18–20,22,23]. Among Norwegian chiropractors, it was more common to work in multidisciplinary settings, often together with other health care practitioners. Nevertheless, clinical settings with direct collaboration with general practitioners and medical specialists were uncommon.

Interdisciplinary communication and meetings were typically found to occur on a weekly basis, however most frequently with physiotherapists and massage therapists. Communication with the patients’ GPs is probably less than optimal, as patient-reports are actually mandatory (by legislation [4]) to the GP. The less than optimal communication from chiropractors to GPs was confirmed in a previous Norwegian study on this topic [11].

Chiropractors in Norway have the right to formally refer patients for medical specialist assessment and to physiotherapists for rehabilitation services. Our results indicate that this generally takes place on a monthly basis, though with wide variation in the frequency. As this legislation was introduced in 2006 [4], it might be too early to determine whether these referral activities are optimal.

Prior to 2006, patients required a referral from a general practitioner in order to be eligible for reimbursement for chiropractic treatment. According to a survey in 1992, at that time, one third of all chiropractic patients were referred from a general practitioner [7]. As this practice was abandoned due to new legislation in 2006 [4], it is not surprising that the present referral rate is much lower. In fact, the official report, in relation to this legislation, documents a reduction of referrals from about 30% to 7% [29]. Similarly, our findings indicate that for the majority of chiropractors, approximately 10% of patients were referred from a general practitioner. This decline is supported by a recent article, indicating that clinical collaboration between Norwegian chiropractors and general practitioners over the last decade was reduced from 35% to 11% [30]. This might seem low, in view of attempts to assimilate chiropractors into the health care sector with this recent legislation. However, one of the main intensions of the new legislation from 2006 was that patients should have direct access to chiropractors without having to consult their GP first. Therefore, the written referrals from GPs to chiropractors are probably at present replaced by more informal recommendations. Hence chiropractors might be unaware that patients have actually been referred.

### Knowledge about clinical guidelines

Most chiropractors reported to be familiar with the Norwegian clinical guidelines of low back pain, though almost 1/4 failed to answer this question. When they were questioned on specific items from this guideline, they agreed with most of them. Some disagreement would be acceptable though, as different guidelines also diverge in their conclusions and only a few key elements can be clearly synthesized [24].

### Comparison with other European countries

There have been practising chiropractors for about a century in both the USA and many European countries. The European Chiropractic Union, an umbrella organization for chiropractic associations in Europe, is actually more than 75 years old [2]. Despite this long-lasting history of chiropractic in Europe, the development, proliferation and maturity of the profession in the various countries have been heterogeneous, primarily as a result of differences in legislation.

There are several demographic and practice-related differences among the chiropractic professions in Europe. For instance, most countries, including Norway, are still dominated by male chiropractors (between 60-80%) as has been the case for more than 20 years in most European countries [13]. In contrast, the male/female ratio is lower in the UK and Denmark [16,17]. Interestingly, both these countries have their own chiropractic education institutions, so perhaps easy access to chiropractic education attracts more female students.

## Conclusion

The Norwegian chiropractic profession is relatively young and practitioners report to be satisfied with their work conditions. There is a clear difference from the earlier practice pattern in that intra- and inter-professional collaboration is more common and it is considered desirable. The profession seems to follow the modern trends in evidence-based practice by using X-rays more sparingly than previously, adhering to guidelines and being positive towards research.
